# Nuclear eDNA estimates population allele frequencies and abundance in experimental mesocosms and field samples

**DOI:** 10.1111/mec.15765

**Published:** 2021-01-12

**Authors:** Kara J. Andres, Suresh A. Sethi, David M. Lodge, Jose Andrés

**Affiliations:** ^1^ Department of Ecology and Evolutionary Biology Cornell University Ithaca NY USA; ^2^ U.S. Geological Survey New York Cooperative Fish and Wildlife Unit Cornell University Ithaca NY USA; ^3^ Cornell Atkinson Center for Sustainability Cornell University Ithaca NY USA

**Keywords:** DNA mixtures, environmental DNA, intraspecific diversity, invasive species, microsatellites, round goby

## Abstract

Advances in environmental DNA (eDNA) methodologies have led to improvements in the ability to detect species and communities in aquatic environments, yet the majority of studies emphasize biological diversity at the species level by targeting variable sites within the mitochondrial genome. Here, we demonstrate that eDNA approaches also have the capacity to detect intraspecific diversity in the nuclear genome, allowing for assessments of population‐level allele frequencies and estimates of the number of genetic contributors in an eDNA sample. Using a panel of microsatellite loci developed for the round goby (*Neogobius melanostomus*), we tested the similarity between eDNA‐based and individual tissue‐based estimates of allele frequencies from experimental mesocosms and in a field‐based trial. Subsequently, we used a likelihood‐based DNA mixture framework to estimate the number of unique genetic contributors in eDNA samples and in simulated mixtures of alleles. In both mesocosm and field samples, allele frequencies from eDNA were highly correlated with allele frequencies from genotyped round goby tissue samples, indicating nuclear markers can be reliably amplified from water samples. DNA mixture analyses were able to estimate the number of genetic contributors from mesocosm eDNA samples and simulated mixtures of DNA from up to 58 individuals, with the degree of positive or negative bias dependent on the filtering scheme of low‐frequency alleles. With this study we document the application of eDNA and multiple amplicon‐based methods to obtain intraspecific nuclear genetic information and estimate the absolute abundance of a species in eDNA samples. With proper validation, this approach has the potential to advance noninvasive survey methods to characterize populations and detect population‐level genetic diversity.

## 1 INTRODUCTION

Environmental DNA (eDNA) approaches are transforming how scientists and resource managers assess the diversity and distributions of organisms (Deiner, Bik, et al., 2017; Taberlet et al., 2012). Using DNA isolated from environmental samples such as ancient and terrestrial sediments, ice cores, and aquatic ecosystems, eDNA methodologies capture the genetic material organisms release into the environment through cells, hair, skin, and faeces (Thomsen et al., 2012; Willerslev et al., 2003, 2007). Such approaches can provide an efficient way to detect species presence/absence (Ficetola et al., 2008; Pilliod et al., 2013), habitat use (Stewart et al., 2017), and relative abundance (Hänfling et al., 2016; Jerde et al., 2011). With greater detection probability and reduced cost over traditional sampling methods, eDNA methods are particularly well‐suited for surveillance of aquatic invasive species, where early detection may be vital for their management or eradication (Dejean et al., 2012; Jerde et al., 2011; Lodge et al., 2016; Vander Zanden et al., 2010). Furthermore, technical advancements in next‐generation sequencing (NGS) methods have led to the development of eDNA metabarcoding, or the simultaneous detection of multiple species with a single molecular marker (e.g., Deiner, Bik, et al., 2017; Kelly et al., 2014; Margulies et al., 2005; Taberlet et al., 2012; Valentini et al., 2016). Environmental DNA can therefore provide information about species distributions, relative abundance, or composition that can be broadly applied in studies of biodiversity, community ecology, and conservation biology (Bohmann et al., 2014; Lodge et al., 2012; Thomsen & Willerslev, 2015).

The majority of eDNA studies to date have assessed biological diversity at or above the species level, with relatively little attention given to intraspecific genetic diversity (Adams et al., 2019; Sigsgaard et al., 2020). However, some recent studies have developed approaches to detect intraspecific genetic variation in the mitochondrial genome from environmental samples (Deiner, Renshaw, et al., 2017; Elbrecht et al., 2018; Parsons et al., 2018; Sigsgaard et al., 2017; Tsuji et al., 2019; Turon et al., 2020). Due to its high copy number per cell, mitochondrial DNA (mtDNA) may occur at higher concentrations in water than nuclear DNA (but see Bylemans et al., 2017; Minamoto et al., 2017; Piggott, 2016), potentially leading to higher detection probability in environmental samples. On the other hand, mtDNA is haploid and nonrecombining so, as a single locus, may be limited in providing the high resolution of genetic variation required for detailed population genetic analyses (Ballard & Whitlock, 2004; Hurst & Jiggins, 2005; Rubinoff et al., 2006; Teske et al., 2018). Expanding eDNA approaches to detect intraspecific variation in nuclear DNA markers such as microsatellites or single nucleotide polymorphisms (SNPs) can therefore enhance our ability to make genetic inferences at the population level.

In this study, we explore the extent to which intraspecific genetic diversity can be detected in eDNA and used to estimate the number of unique genetic contributors to an eDNA sample. As a proof of concept, we use nuclear microsatellite markers and NGS methods to characterize population allele frequencies and estimate the absolute abundance of round gobies (*Neogobius melanostomus*) using eDNA samples from experimental mesocosms and in a field‐based trial. The round goby, a fish species native to the Ponto‐Caspian region, was initially introduced to North America via ballast water in 1990 and has since spread throughout the Laurentian Great Lakes (Charlebois et al., 1997; Jude et al., 1992; Schaeffer et al., 2005). More recently, round gobies have spread to inland lakes and rivers, where they can cause native species declines through competition, predation, and contaminant cycling (Janssen & Jude, 2001; Kornis et al., 2012; Krakowiak & Pennuto, 2008). Due to the short time interval between arrival and establishment, round gobies present a high invasion risk even at low densities (Vélez‐Espino et al., 2010), and control strategies may require information on species abundance due to the rapid decline in the success of eradication efforts as invasive populations grow and spread (Vander Zanden et al., 2010). Thus, the development of eDNA methods to quantify species abundance at the invasion front could lead to improved management strategies for this invader.

Several previous efforts to assess species abundance with eDNA have used correlative relationships between eDNA concentration and indices of species abundance or biomass (e.g., Kelly et al., 2014; Pilliod et al., 2013; Takahara et al., 2012). While these methods can provide an index of relative abundance, their accuracy and precision with respect to absolute species abundance has been difficult to establish, and such correlative relationships can be heavily impacted by taxon‐specific amplification biases (Kelly et al., 2019) or local biotic and abiotic factors influencing the amount of DNA shed by an organism (Barnes & Turner, 2015). For instance, the production rate of eDNA can vary with an organism's size, behaviour, or metabolism, all of which may vary across a range of abiotic conditions (Klymus et al., 2015; Lacoursière‐Roussel et al., 2016; Maruyama et al., 2014; Takahara et al., 2012). The difficulty in obtaining robust quantitative measurements of eDNA production among individuals and its relationship to the amount of DNA in an eDNA sample currently limits our ability to reliably link measurements of eDNA concentration to species abundance, density, or biomass (Iversen et al., 2015).

In contrast to correlative relationships between eDNA concentrations and relative species abundance, DNA mixture estimators take a radically different approach to estimating absolute abundance in a sample (Sethi et al., 2019). Originally developed in criminal forensics, DNA mixture estimators provide an inferential framework that uses the genetic signature of mixtures to estimate the number of unique genetic contributors in a mixture of DNA based on population allele frequencies and the number of unique alleles identified (Curran et al., 1999; Weir et al., 1997). While these estimators have previously been applied to tissue‐based mixtures of DNA for diet analysis (Sethi et al., 2019), environmental samples can also contain DNA from multiple individuals. If intraspecific genetic diversity can be detected eDNA, mixture estimators may therefore provide a means of estimating the number of contributors to environmental samples that relies on the detected presence of haplotypes or alleles rather than eDNA concentrations.

Here, we applied DNA mixture estimators to eDNA using species‐specific nuclear genetic markers we developed for round gobies. We first assessed the similarity of allele frequencies from eDNA and individually genotyped individuals in experimental mesocosms to evaluate the extent to which alleles derived from round goby tissues are represented in sequence data recovered from eDNA. We then used a likelihood‐based DNA mixture model to estimate the number of genetically unique individuals contributing genetic material to each eDNA sample. Finally, we tested the ability of the DNA mixture estimator to accurately estimate the number of unique genetic contributors in simulated combinations of up to 58 individuals.

## 2 MATERIALS AND METHODS

### 2.1 Microsatellite characterization and multiplex assay development

Genomic DNA (50–100 ng) from a pool of three round goby (*Neogobius melanostomus*) individuals collected from Cayuga Lake, New York, USA was endonuclease‐digested with *AluI*, *RsaI*, and *Hpy166II*. The digestions were pooled for subsequent adenylation with Klenow (exo‐) and dATP, and the resulting products were ligated to an Illumina Y‐adaptor sequence using T4 DNA ligase in the presence of 1 mM ATP. Genomic fragments containing repeats were captured by hybridization to biotinylated repeats and streptavidin‐coated magnetic beads followed by amplification with Platinum Taq DNA polymerase and indexing with Illumina primers (one universal primer and one index primer). PCR products were quantified with a Qubit 2.0 fluorometer (Life Technologies, Carlsbad, CA), verified by electrophoresis on a 1.0% agarose gel, and size‐selected (300–600 base pairs [bp]) with Agencourt AMPure XP beads (Beckman Coulter, Indianapolis, IN). The “design primers” function of MSATCOMMANDER 1.0.3 (Faircloth, 2008; Rozen & Skaletsky, 2000) was then used to create a library of microsatellite tetramer repeats based on the number of motif repeats (10–24) and PCR product length (410–440 bp).

Primer specificity was inspected using NCBI Primer Blast, where no other species were detected as matches to the designed primer pairs. Forward and reverse primers (range 20–24 bp) for 43 loci were ordered from Integrated DNA Technologies (http://www.idtdna.com) and tested for functionality in single reactions using genomic DNA extracted from the tissue of three round gobies. Following exclusion of primers with complementary sequences or suboptimal PCR amplification, 35 microsatellite loci remained (Table S1).

Microsatellite loci were grouped into seven multiplex PCR assays, with each multiplex containing 4–6 primer pairs (Table S1). We tested the performance of each multiplex in a PCR containing 1 µl (20–30 ng) of round goby genomic DNA, 1 µl of primer pairs in equimolar concentrations (2 µm), and 5 µl of Qiagen Multiplex PCR Master Mix (Qiagen Inc.). The program for multiplex PCR is as follows: initial denaturation at 95°C for 15 min followed by 35 cycles of 94°C for 30 s, 59°C for 90 s, and 72°C for 90 s. Gel electrophoresis in 1% agarose stained with ethidium bromide confirmed the presence of PCR products within the expected size range for all multiplexes.

### 2.2 Mesocosm experiment

We collected live round gobies (*n* = 58) from a site on Cayuga Lake via beach seining and placed them in one of 12 experimental mesocosms containing 12 L of aged room temperature tap water. Each mesocosm treatment was conducted in triplicate and contained round gobies (approximately 7–12 cm length) at densities of one, three, five, or 10 individuals. An additional round goby was erroneously added to a single replicate of the *n* = 10 treatment to total 11 individuals (labelled mesocosm 10c), but is hereafter grouped into the density treatment of 10 individuals. Two additional mesocosms served as negative controls (mesocosms with aged room temperature tap water only). After 1 h, round gobies were removed from the mesocosms and euthanized with MS‐222 according to the Cornell IACUC Animal Care and Use Procedure (ACUP 306.02). Tissues were sampled from caudal fins of each individual and DNA was extracted with a DNeasy Blood and Tissue extraction kit (Qiagen Inc.) following the manufacturer's protocols. Following the removal of all fish from the mesocosms, duplicate 2 L water samples were collected from each mesocosm in sterilized wide‐mouth Nalgene plastic bottles and stored on ice until vacuum‐filtration through a cellulose nitrate membrane filter (47 mm diameter, 1 µm pore size). Filters were immersed in 700 µl Longmire's solution (100 mm Tris, 100 mm EDTA, 10 mm NaCl, 0.5% SDS, 0.2% sodium azide) and stored at –20°C until DNA extraction. Environmental DNA was extracted from filters following a modified protocol from the DNeasy Blood and Tissue extraction kit (Qiagen Inc.) as in Spens et al. (2017). To minimize contamination, eDNA sample filtration and pre‐PCR laboratory protocols were carried out in separate rooms within dedicated pre‐PCR facilities, and stringent precautions were followed according to Goldberg et al. (2016). Round goby tissues were handled and processed in a separate facility. All reusable equipment including collection bottles, forceps, and the vacuum filtration apparatus was cleaned between samples by soaking in a 50% commercial bleach solution, rinsing in DI water, and treating under UV bulbs for 30 min each. In addition to the two field controls described above, one filtration blank and one PCR blank served as negative controls.

### 2.3 Field trial

To determine the feasibility of estimating population allele frequencies from eDNA samples in a field‐based setting, we collected eDNA samples and additional round gobies (*n* = 15) from another site on Cayuga Lake (c. 20 miles away from the site of round goby collection for the mesocosm experiment; Figure S1A). Sampling was conducted during the summer months when round goby densities peak in nearshore waters, and density estimates from a previous study using benthic videography and direct observation report round goby densities of 0.34 fish/m^2^ in this section of the lake (Andres et al., 2020). We confirmed the round gobies collected from the two sites in Cayuga Lake are panmictic using genotyped tissue samples from both sites and the “find.clusters” function of the ADEGENET package in R version 3.5 (Jombart, 2008; R Core Team, 2016), where a single cluster (k) exhibited the lowest Bayesian Information Criterion (BIC; Figure S1B). Thus, we consider all 73 round gobies (58 in the mesocosm experiment and 15 in the field trial) when estimating tissue‐based population allele frequencies in the field trial. To sample eDNA, three 2 L water samples were collected from shoreline locations approximately 50 m apart in sterilized wide‐mouth Nalgene plastic bottles. A negative field control of 2 L of distilled water was also collected at the site. Water filtration, tissue sampling, and DNA extraction protocols were identical to those described for the mesocosm experiment above.

### 2.4 Library preparation and MiSeq sequencing

Microsatellite loci were amplified from eDNA and tissue samples in separate reactions using multiplex PCR methods described above, with the number of PCR cycles increased to 45 for eDNA samples due to low template DNA concentrations. Three PCR replicates were performed for each of the three eDNA samples from the field trial. Products from all seven multiplexes were pooled from each sample in equal volumes (5 µl each) and uniquely barcoded in a second‐stage PCR using Illumina Nextera XT tags. Each 20 µl second‐stage PCR included 2 µl pooled PCR product diluted 1:1 with molecular H_2_O, 4 µl 5× HF buffer, 0.4 µl 10 mm dNTPs, 0.1 µl OneTaq DNA polymerase, 0.4 µl each of 10 µm Nextera Index Primer 1 (N701–N728) and Nextera Index Primer 2 (N502–N521). One library was constructed from the pooled PCR products for all tissue and eDNA samples in the mesocosm experiment, while another library was constructed from the tissue and eDNA samples from the field trial. DNA libraries were purified with Agencourt AMPure XP beads and the concentration of each library was estimated using the Qubit dsDNA High‐Sensitivity Kit and Qubit 2.0 fluorometer. The libraries were diluted to 4 nm with PCR‐grade water and paired‐end sequenced on an Illumina MiSeq sequencing platform (Illumina, San Diego, CA) with the MiSeq v2 500 bp kit (PE 2 × 250 bp) by Cornell University's Institute of Biotechnology Genomics Facility.

### 2.5 Bioinformatic analysis

Demultiplexed reads from each Miseq run were processed with trimmomatic v0.39 (Bolger et al., 2014) to remove adapter sequences. We then ran a custom Perl script to extract forward and reverse reads and assign them to each locus as described in D’Aloia et al. (2017). The script includes the following steps: (i) trim low‐quality reads with Phred scores less than 20; (ii) create contigs from overlapping paired‐end reads with a minimum overlap of at least 20 bp and mismatch rate of less than 0.05; (iii) identify and sort reads corresponding to each locus using the forward primer; (iv) collapse identical reads (100% identity) for each sample; and (v) collapse reads across all samples. To filter out most PCR artifacts and paralogues while retaining true microsatellite repeats and SNPs, we required 90% of the first 40 bp of a read to align with and match the reference contig constructed from the most common allele at each locus across all of the samples. We determined the multilocus diploid genotype for each round goby tissue sample based on the allele with the highest read count at each locus. Individuals were considered heterozygous at a locus if at least 20% of the reads corresponded to a second allele, and only alleles with a read depth of at least 10 reads per individual were considered (as in D’Aloia et al., 2017). Following individual genotyping, we excluded two poorly amplified loci and five potentially paralogous loci exhibiting significant deviations from Hardy‐Weinberg equilibrium (Paradis, 2010) and heterozygote excess. The remaining 28 loci were used in further analyses (Table S1).

For eDNA samples, we excluded alleles with fewer than 10 total reads in each sample and scaled read counts to 100 reads per sample to account for differences in read depth. To further filter out potentially erroneous sequence data arising from PCR stutter and sequencing error, we removed alleles below 1% frequency in each eDNA sample from analysis. Due to low variation in read depth and allele frequencies between duplicate mesocosm eDNA samples (Figures S2–S3), we pooled the scaled reads from the two eDNA samples for each mesocosm. We also pooled the scaled reads from the three replicate eDNA samples from the field trial. eDNA allele frequencies were then estimated as the read frequencies of alleles in each mesocosm and in the field eDNA sample. Thus, while allele frequency estimations in tissue samples are derived from genotyped individuals, allele frequency estimations in eDNA samples are taken directly from sequence read frequencies.

### 2.6 Comparison of genotyped individuals and eDNA samples

All further analyses were performed in R version 3.5 (R Core Team, 2016). To determine the similarity between allele frequencies derived from eDNA reads and genotyped tissues in the mesocosm experiment, we combined allele frequencies across all 12 mesocosm eDNA samples and evaluated the correlation between eDNA allele frequencies and tissue allele frequencies for all alleles across all loci, as well as on a per‐locus basis. We further examined the similarity between eDNA‐based and tissue‐based allele frequencies in corresponding mesocosms by conducting a principal components (PC) analysis on the scaled and centred allele frequencies from eDNA reads and genotyped individuals. Subsequently, we constructed a Euclidean distance matrix for all samples using principal components values along all PC axes described above as inputs. For the field trial, we evaluated the correlation between allele frequencies determined from the eDNA samples collected from Cayuga Lake and from the 73 genotyped round gobies.

### 2.7 DNA mixture contributor estimation

To estimate the number of unique genetic contributors to a DNA mixture (e.g., the number of individuals captured in each eDNA sample), we implemented a likelihood‐based model described in Sethi et al. (2019). At each locus *j*, the model estimates the likelihood that a proposed number of diploid contributors, *x*, produces the observed set of *n* alleles, $A=\left\{a_{1},\ldots ,a_{n}\right\},$ given a set of associated population allele frequencies, $p=\left\{p_{1},\ldots ,p_{n}\right\}$, using the following equation:

(1) $$L_{j}\left(x|A,p\right)=\sum_{d_{1=0}}^{d}\sum_{d_{2}=0}^{d‐d_{1}}\ldots \sum_{d_{n‐1}}^{d‐d_{1}‐\ldots ‐d_{n‐2}}\left[\left(\frac{\left(2x\right)!}{\prod_{i=1}^{n}g_{i}!}\right)\prod_{i=1}^{n}p_{i}^{g_{i}}\right]$$

This equation accounts for all of the combinations of alleles that may arise in a mixture due to redundancy within or among individuals, where d=2x‐n is the total number of “masked” alleles calculated as the difference between the total number of alleles present for x diploid organisms and the total number of unique alleles observed in the mixture genotype, and $g_{i}$ is the total number of copies of allele $a_{i}$ truly present in the mixture plus any masked copies of the allele $d_{i}$, with $\sum_{i=1}^{n}g_{i}=2x$. As in Sethi et al. (2019), we calculated this likelihood with custom R scripts using a numerically equivalent but more computationally efficient form of Equation 1 derived by Weir et al. (1997).

The estimated number of individuals contributing to the DNA mixture is therefore identified as the maximum likelihood estimate of the number of contributors given the product of this likelihood across all loci:

(2) $$\max_{x}\prod_{j}L_{j}\left(x|A,p\right)$$

For the mesocosm samples, we applied this equation to pooled individual genotypes and eDNA mixtures from each mesocosm with a proposed number of contributors (1–100), where the set of observed alleles A was determined per mesocosm and the population allele frequencies *p* were estimated directly from the 58 genotyped individuals used in the experiment. To evaluate the sensitivity of the contributor estimation to false alleles and allelic dropout, we filtered eDNA sequence reads according to a succession of increasingly strict thresholds, or frequencies below which reads were removed (0.001, 0.01, 0.1). Due to variation in the number of alleles present at each locus (Table S1), we also filtered reads using variable thresholds according to per‐locus allelic richness, where the threshold decreased from 0.1 to 0.001 as the number of alleles at a locus increased. We repeated the contributor estimations using the allele frequencies combined across all eDNA samples to represent population allele frequencies *p*. Bias in the contributor estimation (estimated # contributors ‐ true # contributors) was calculated for each eDNA‐based and tissue‐based DNA mixture.

To assess the performance of the contributor estimation on eDNA samples representing a greater number of individuals, we applied the maximum likelihood estimator to simulated mixtures of up to our total sample of 58 round gobies in the mesocosm experiment. Using a bootstrapping procedure, we combined eDNA read counts from mesocosms in simulated mixtures ranging from 2–12 mesocosms per draw. We estimated the number of genetic contributors to mixtures with 1,000 bootstrap replicates at fixed thresholds and a variable threshold based on allelic richness as described above.

We also applied the contributor estimation to each eDNA sample from the field trial, where the set of observed alleles A was determined from each eDNA sample and population allele frequencies *p* were estimated from the 73 genotyped individuals used in the experiment. We repeated the contributor estimations with allele frequencies combined across the three replicate eDNA samples taken from the field used to represent population‐level allele frequencies *p*.

## 3 RESULTS

### 3.1 Sequencing and genotyping

The full data set contained 47,920,390 reads, of which 35,583,440 remained after demultiplexing and trimming adapters. Following exclusion of alleles below the minimum read depth of 10 reads, the target loci were not identified in any of the negative control blanks from the field, extraction, or amplification processes, indicating there was no detectable cross‐contamination. Round goby tissue samples exhibited a high total read depth per sample (mean = 45,534 reads, SD = 19,958; Figure S4A) and total read depth per locus (mean = 1,626 reads, SD = 1,714, Figure S4B). All individuals were genotyped at ≥26 of the 28 loci in all individuals (i.e., fewer than two loci per sample were considered missing data in our pipeline). All microsatellites were multiallelic with an average of 9.4 alleles per locus (range: 2–21 alleles per locus) among the 73 round gobies comprising the sample population (Table S1). Microsatellite loci were successfully amplified in all eDNA samples from mesocosms containing fish with an average total read depth of 37,151 reads per sample (SD = 9,161) and average read depth of 1,327 reads per locus (SD =1,393). The average per‐locus read depth and total read depth did not vary across mesocosm densities (Figure S5), indicating round goby density did not have an impact on sequence recovery in the mesocosm experiment. Read depths were lower in eDNA samples from the field trial, with an average total read depth of 4,305 reads per sample (SD =3,796; Figure S4A) and 154 reads per locus (SD =283; Figure S4B).

### 3.2 Comparison of genotyped individuals and eDNA samples

**FIGURE 1:**
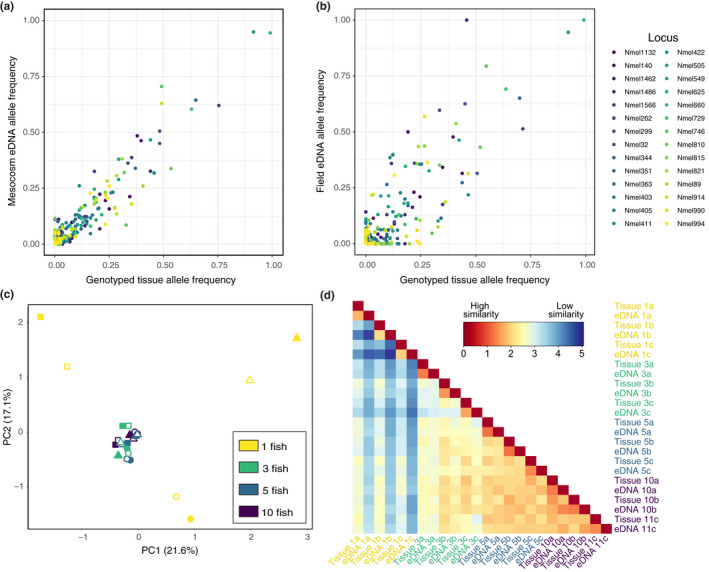
(a) Correlation between eDNA‐derived and tissue‐derived allele frequencies for all alleles across 28 loci in the mesocosm experiment. (b) Correlation between eDNA‐derived and tissue‐derived allele frequencies for all alleles across 28 loci in the field trial. (c) PCA of allele frequencies across 28 loci for round goby tissue samples (filled symbols) and eDNA samples (hollow symbols) from 12 mesocosms varying in round goby density. Colours represent mesocosm density treatments (1, 3, 5, or 10 fish) and symbols represent treatment replicates. (d) Heatmap of the pairwise Euclidean distances across all PC axes of allele frequencies from mesocosm eDNA and tissue samples, with blue colours indicating far distances (low similarity) and red colours indicating close distances (high similarity). Samples are arranged in pairs (eDNA/tissue samples) from each mesocosm, with colours representing mesocosm density treatments and letters (a, b, or c) representing treatment replicates

In the mesocosm experiment, allele frequencies from eDNA sequence reads across all mesocosms closely resembled allele frequencies from the 58 genotyped individuals (Pearson's correlation coefficient *r* = 0.95 across all loci, range *r* = 0.88–1.00 per locus; Figure 1a). Principal component analysis results showed high similarity between eDNA samples and genotyped individuals in each mesocosm, where eDNA samples clustered tightly with the individuals from the associated mesocosm (Figure 1c). Across all PC axes, the pairwise Euclidean distance was noticeably smaller within a mesocosm than between mesocosms (Figure 1d). eDNA and tissue sample pairs were most differentiated from other samples when derived from mesocosms with single round gobies, becoming more genetically similar to other mesocosms as the number of round gobies per mesocosm increased.

Allele frequencies from eDNA samples and genotyped individuals were also highly correlated in the field trial, with a Pearson's correlation coefficient of *r* = 0.84 across all loci (range *r* = 0.41–1.00 per locus; Figure 1b). Several alleles at low frequency in the population were not recovered by eDNA samples, with only 121 of 253 total alleles identified from genotyped individuals occurring in at least one of the three eDNA samples. However, all alleles with a frequency >0.24 in the 73 genotyped individuals were recovered by at least one of the three eDNA samples, and alleles not detected with eDNA occurred at low frequencies in the population (mean =0.03, SD = 0.04). On the other hand, eDNA samples also identified several alleles not documented in the genotyped individuals, albeit at low read frequencies (mean = 0.02, SD = 0.02). Such alleles may represent true low‐frequency alleles not included in the genotyped individuals or may be the product of erroneous sequences.

### 3.3 Contributor estimation

**FIGURE 2:**
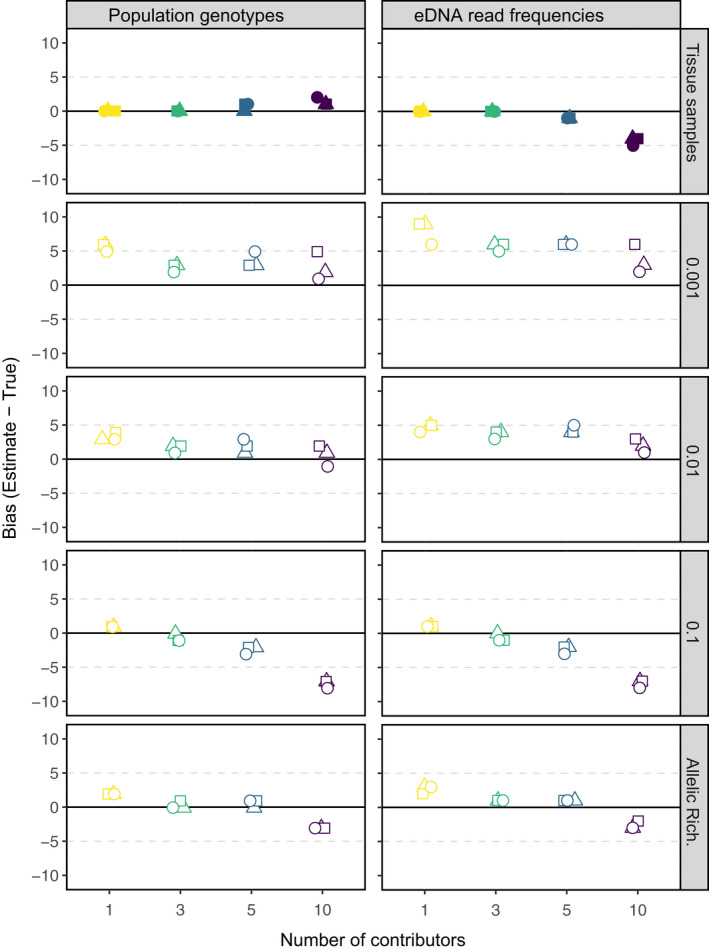
Bias of the contributor estimation using genotypes from round goby tissue samples (filled symbols) and eDNA samples (hollow symbols) across mesocosm treatments of round goby density (1, 3, 5, or 10 fish). The population allele frequencies for mixture estimation input were derived from 58 genotyped individual round gobies (left) or from eDNA read frequencies combined across all mesocosms. Symbols represent treatment replicates and panels indicate fixed threshold frequencies below which sequence reads were removed (0.1, 0.01, 0.001) or a variable threshold based on per‐locus allelic richness (Allelic Rich.)

Estimates of the number of genetic contributors in mesocosms using observed alleles from genotyped tissue samples were within ±2 contributors at all round goby densities when population‐level allele frequencies were specified using genotyped tissues and in mixtures of up to five individuals when allele frequencies were specified from eDNA read frequencies (top panel, Figure 2). When estimating the number of genetic contributors using observed alleles from eDNA samples, patterns of bias emerged across frequency thresholds below which reads were removed (0.001, 0.01, 0.1) regardless of how population‐level allele frequencies were characterized. The contributor estimation was positively biased the lowest thresholds (0.001 and 0.01) across all mesocosm densities with the exception of the 10‐individual mixtures using population allele frequencies from genotyped individuals, where estimates were within ±1 genetic contributor. Contributor estimations were also within ±1 contributors in mesocosms with one or three round gobies at the highest threshold (0.1), while negative bias was more apparent in mesocosms with five or 10 individuals at this threshold (Figure 2). Across all mesocosm densities, the variable threshold based on allelic richness outperformed all other thresholds (maximum bias = +5 associated with a 10‐individual mixture using population allele frequencies from eDNA reads). Thus, adjusting the threshold according to the per‐locus allelic richness provides the most accurate estimate of absolute abundance in a mixed‐DNA sample.

At the lowest threshold (0.001), contributor estimations of eDNA samples exhibited a positive bias across all densities, indicating the presence of false positive alleles in eDNA reads. However, the lack of cross‐contamination in the negative control samples indicates the false positive alleles probably arose from artifacts introduced during PCR or sequencing, rather than cross‐contamination between eDNA and tissue samples. The issue of positive bias in contributor estimations was more prevalent when population allele frequencies were specified using eDNA read frequencies. Nonetheless, patterns of bias and estimates of the number of genetic contributors in mesocosms were similar regardless of whether the input population allele frequencies were characterized using eDNA read frequencies or tissue‐based allele frequencies (Figure 2). Thus, under controlled conditions, genotyped individuals may not be required to obtain reliable estimates of the absolute abundance of a species in eDNA samples.

**FIGURE 3:**
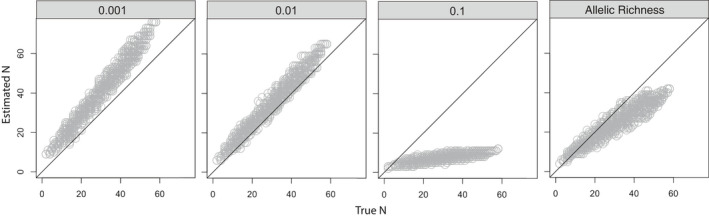
Estimated number of individuals contributing to simulated eDNA mixtures (range 2–58 individuals) using alleles from 1,000 simulated eDNA mixtures generated by constructing combinations of up to 12 mesocosms. Panels correspond to fixed threshold frequencies below which sequence reads were removed (0.001, 0.01, 0.1) or a variable threshold based on per‐locus allelic richness. Diagonal lines represent a 1:1 relationship (i.e., zero bias for mixture contributor estimates)

In simulated mesocosm mixtures, the number of individuals could be reasonably estimated in mixtures up to 58 individuals, although bias associated with threshold values were apparent (Figure 3). The highest threshold (0.1) often exceeded the allele frequencies for all but the most common alleles, resulting in a negative bias in the contributor estimation. At this threshold, the maximum contributor estimation peaked at around 15 individuals, even at simulated densities >50 individuals. On the other hand, filtering the eDNA reads according to lower thresholds (0.01, 0.001) appeared to overestimate the number of contributors across all densities. The variable threshold based on allelic richness showed lower variation in the estimated number of contributors and performed well for all but the largest numbers of contributors, where a negative bias occurred. Thus, while our 28‐locus panel was able to resolve mixtures of up to 58 individuals, filtering decisions can have a large effect on the estimated number of contributors in eDNA samples.

In the field trial, the contributor estimation resulted in an estimated five, three, and three genetically distinct individuals captured by the three replicate eDNA samples when population‐level allele frequencies *p* were estimated from the 73 genotyped individuals. However, because the contributor estimation calculations only consider alleles from the specified population‐level allele frequencies, this is probably an underestimate as we did not recover several low‐frequency alleles from the genotyped tissues in the eDNA samples. When population‐level allele frequencies were specified using the combined reads from the three replicate eDNA samples, an estimated 13, 7, and six genetically distinct individuals contributed to the mixture of DNA from each sample, respectively.

## 4 DISCUSSION

Estimating the genetic diversity and abundance of a species provides insights into a wide range of ecological and evolutionary processes and may have important implications for conservation management opportunities. While analysis of eDNA is a well‐established approach for detecting species, it also holds potential to detect genetic diversity within species (Adams et al., 2019; Sigsgaard et al., 2020). With this study, we use eDNA and NGS methods to detect intraspecific genetic diversity of an aquatic invasive species by recovering microsatellite allele frequencies that are similar to those derived from genotyped tissue samples in experimental mesocosms and in field‐based eDNA collections. Using DNA mixture analyses, we estimated the number of genetic contributors of the target species within environmental samples, demonstrating the ability to use intraspecific genetic information to estimate the number of individuals captured in an eDNA sample. Although technical challenges regarding the parsing out of sequencing noise from low‐abundance alleles in eDNA samples remain, this study experimentally validates the use of nuclear microsatellites to estimate population‐level allele frequencies and absolute abundance of aquatic species using eDNA methods, a requisite step toward population‐level inferences using nuclear eDNA.

To date, studies using eDNA approaches to characterize intraspecific genetic variation in aquatic species have been limited to a single locus in the mitochondrial genome (Elbrecht et al., 2018; Parsons et al., 2018; Sigsgaard et al., 2017; Tsuji et al., 2019; Turon et al., 2020). The expansion of eDNA approaches to target multiallelic nuclear DNA markers could allow for the detection of robust higher‐resolution population‐level genetic information from water samples, as is common practice in contemporary tissue‐based population genetics studies. In controlled mesocosms, we document microsatellite allele frequencies from eDNA closely resembled tissue‐based allele frequencies across all mesocosms and on a per‐mesocosm basis, although our approach exhibited decreased sensitivity in genetically distinguishing mesocosms from one another at high round goby densities (Figure 1c–d). Because we used round gobies derived from a single population source, this is to be expected. We also demonstrate reasonably accurate allele frequency estimates from eDNA samples collected in natural conditions in a field trial, albeit with reduced detection of rare alleles in the population. Such eDNA‐based estimates of population‐level allele frequencies could potentially be used in population genetic inferences and demographic analyses using eDNA sampling methods. However, because eDNA samples contain a pool of DNA from many individuals, this approach is unable to determine multilocus genotypes or assign genotypes to individuals, and methods designed to analyse population genetics using individual genotypes will need to be adapted into an eDNA framework. Theoretical and analytical frameworks for estimating population genetic parameters from pooled tissue samples of many individuals (Pool‐seq) have already been developed (Boitard et al., 2013; Gautier et al., 2013; Hivert et al., 2018), and similar frameworks may be useful for eDNA‐based population genetics. As emphasized in Sigsgaard et al. (2020), however, such frameworks may need to account for additional potential sources of bias affecting the precision of population allele frequency estimates from eDNA, including variation in the number of individuals sequenced, unequal contributions of DNA from individuals, and variation from library preparation and sequencing.

Detecting intraspecific genetic variation in eDNA samples is also useful for estimating the number of genetically distinct individuals detected in a sample, which may be advantageous over approaches using DNA concentrations to predict species abundance or biomass. With the number of loci used in this study, the number of genetic contributors in simulated mixtures of up to 58 individuals could be resolved. While contributor estimations at the highest allele frequency threshold provided the most accurate estimates at low round goby densities in the mesocosm experiment, they were insufficient in resolving high numbers of round gobies, probably due to the removal of true low‐frequency alleles below the threshold limits. In contrast, low thresholds sufficiently resolved the number of contributors at high round goby densities but erroneously inflated the number of contributors at low densities due to the introduction of false alleles. We therefore recommend bioinformatic filtering based upon moderate thresholds or variable thresholds associated with locus‐specific allelic richness in future applications of DNA mixture analysis. However, we also caution future studies to further investigate the possible impacts of false alleles and allelic dropout on contributor estimations, particularly in field‐based settings where false alleles are more difficult to distinguish from true low‐abundance alleles and detection of rare alleles may be low. Because low‐frequency alleles provide strong information on the number of individuals present in a sample (Sethi et al., 2019), efforts to maximize the recovery of low‐frequency alleles through optimization of field and laboratory protocols may be required to obtain accurate estimates of the number of individuals captured in eDNA samples. Additionally, applications of error‐correction algorithms and denoising procedures may be required to aid in the detection and removal of erroneous sequences while retaining true low‐frequency alleles (Elbrecht et al., 2018; Tsuji et al., 2019; Turon et al., 2020).

Future eDNA studies may consider the use of single nucleotide polymorphisms (SNPs) as a target nuclear marker, as they are an abundant and widespread form of variation throughout the genome of most species (Morin et al., 2004). However, because the inferential power of the DNA mixture model is limited by the number of recovered alleles, much larger marker panels of biallelic SNPs will be needed to resolve eDNA mixtures into the number of genetic contributors, particularly as the number of contributors grows (Sethi et al., 2019). Rather than targeting single SNPs, a potential solution may be to target several SNPs occurring in the same genomic region that can be jointly genotyped (Kidd et al., 2013). Such multiallelic “microhaplotype” markers have high per‐locus information content in a small length of DNA and may reduce the potential for analysis errors that arise when targeting microsatellites including PCR stutter and allelic dropout.

Although our approach demonstrates promise for future applications of noninvasive population genetic sampling using nuclear eDNA, the controlled settings of our mesocosm experiments and limited spatial and temporal scale of the field trial may not reflect the complexity of in situ conditions. Thus, several obstacles may need to be addressed before this approach can be broadly applied in field‐based settings. For instance, although round gobies may exhibit localized hotspots of high density, the average density of round gobies in occupied habitats of Cayuga Lake (1.82 fish/m^2^) is lower than in our mesocosm experiments (Andres et al., 2020), and read depths we observed in mesocosm eDNA samples may not be achievable in field settings. Indeed, even with targeted eDNA sampling in areas of high expected round goby densities, read depths in eDNA samples from the field trial averaged 4,305 reads per sample, which is much lower than reported in other eDNA studies using targeted field sampling and markers in the mitochondrial genome (e.g., average 263,111 reads per sample at sites where whale sharks were reported, Sigsgaard et al., 2017; average 237,434.5 reads per sample taken from harbour porpoise fluke prints, Parsons et al., 2018). To ensure genetic data obtained from eDNA samples sufficiently reflects the genetic diversity of the population of interest when targeting loci in the nuclear genome, efforts to evaluate the limit of detection and optimize field and laboratory strategies to achieve sufficient eDNA copy numbers may be required (Adams et al., 2019; Sigsgaard et al., 2020).

Mesocosm conditions also lacked the biophysical complexity inherent in natural systems, where many other particles and organisms are present and contributing to eDNA samples (Barnes & Turner, 2015). PCR inhibition from nontarget particles may restrict accurate molecular identification of alleles, particularly when coupled with low eDNA concentrations of target species DNA (Hunter et al., 2019). Importantly, if closely related nontarget species are found in the sampled habitats, primer specificity must be thoroughly tested to ensure DNA from co‐occurring nontarget species is not amplified. While no congeners of the round goby are found in North America, the freshwater tubenose goby (*Proterorhinus semilunaris*, formerly known as *P*. *marmoratus*; Stepien & Tumeo, 2006) is found throughout the Great Lakes. Although we tested primer specificity in silico using DNA databases, in vitro testing using tissue‐derived DNA from nontarget species may also be required if reference sequence data is lacking for closely related co‐occurring species.

With proper validation and appropriate analytical frameworks, eDNA‐based population genetics has the potential to enhance the use of eDNA methods in conservation and management of species. For example, preventing the spread and minimizing the undesirable impacts of invasive species will require effective monitoring of non‐native populations, including evaluating population‐level genetic variation and population size at the sites of initial colonization (Le Roux & Wieczorek, 2009). With further development, this method might someday inform management strategies at early stages in the invasion process when eradication efforts are most likely to be successful in preventing proliferation and future spread (Leung et al., 2002; Lodge et al., 2016). This approach may also be beneficial for monitoring species where small population sizes, expansive or complex habitats, elusive behaviour, or a desire to minimize invasive sampling can prevent effective population assessments. For instance, Parsons et al. (2018) used eDNA approaches to generate mitochondrial sequence data in a highly elusive marine mammal where physical tissue‐based sampling presents logistical challenges and limits the detection of population genetic structure. The high sensitivity of eDNA methods and relative ease of sample collection therefore present a noninvasive and potentially cost‐effective opportunity to study the population genetics of aquatic organisms for which traditional sampling is difficult or impossible.

As with other eDNA methods such as DNA metabarcoding, the approach developed here is likely to complement, rather than replace, existing methods of evaluating intraspecific diversity in population genetics studies (Yoccoz, 2012). Indeed, developing species‐specific panels of microsatellite DNA markers requires sufficient DNA sequence data for the species of interest, and optimization of multiplex PCR requires testing on tissue‐derived DNA samples. Estimating the number of contributors to eDNA samples also requires an assessment of population allele frequencies, an estimate that may be derived from tissue‐based genotyping of the population of interest. However, we demonstrate that under controlled experimental conditions, population allele frequencies from eDNA read frequencies are highly correlated with allele frequencies from genotyped individuals and contributor estimations are similar regardless of where the population allele frequencies are derived. Estimating the number of contributors in eDNA samples may therefore be feasible even in the absence of population‐level sequence information from tissue samples.

As the time and costs associated with obtaining and analysing molecular data continue to decline, eDNA methodologies may become an increasingly effective approach for detecting and quantifying the presence of invasive, rare, or threatened species. Moreover, with the recent expansion of eDNA approaches into studies of intraspecific diversity, the scope of eDNA applications has broadened to population‐level inferences. With this study, we demonstrate the advancement of eDNA approaches to encompass genetic markers in the nuclear genome, with implications for future studies of population genetics using next‐generation sequencing of environmental samples. By incorporating DNA mixture analyses into a nuclear genome‐based eDNA framework, we estimate the number of unique contributors to eDNA samples, providing the first steps to a potential alternative to correlation‐based estimates of species abundance using DNA concentration. Furthermore, we demonstrate the ability to obtain population‐level genetic information from nuclear eDNA, supporting the potential for future assessments of population genetics from environmental samples. Provided further validation and optimization in field‐based settings, such an advancement could transform the ways in which we obtain population‐level genetic information on species of conservation or management concern.

## AUTHOR CONTRIBUTIONS

All authors conceived and designed the study, interpreted results, and contributed to writing the manuscript. K.J.A. conducted the study and collected specimens. K.J.A., and J.A completed laboratory work and analysed the data. All authors approved the manuscript for publication.

## Supporting information

Figure S1‐S5

## Data Availability

Illumina MiSeq raw sequence data are uploaded to NCBI’s Sequence Read Archive (BioProject ID: PRJNA680257). Microsatellite primers are available in Table S1. All scripts used in the data processing and analysis are available on GitHub (https://github.com/karaandres/eDNA_goby_mesocosms).
