# Supplementary material for: Nuclear eDNA estimates population allele frequencies and abundance in experimental mesocosms and field samples
Source: Mol Ecol. 2021 Jan 12;30(3):685–97. doi: 10.1111/mec.15765 (PMC7898893; doi:10.1111/mec.15765)
Supplement: Supplementary file 1 — Figure S1‐S5 [file MEC-30-685-s001.docx]

**Supplemental Information for:**

**Nuclear eDNA estimates population allele frequencies and abundance in experimental mesocosms and field samples**

K.J. Andres, S.A. Sethi, D.M. Lodge, and J. Andres

**Table of Contents:**

| **Table S1.** Summary information for 35 autosomal microsatellite loci. | Page 2 |
| --- | --- |
| **Figure S1.** Sites of mesocosm/field collections of round gobies and BIC versus number of clusters. | Page 3 |
| **Figure S2.** Correlation of allele read depth between duplicate eDNA samples per mesocosm. | Page 4 |
| **Figure S3.** Correlation of allele read frequencies between duplicate eDNA samples per mesocosm. | Page 5 |
| **Figure S4.** Mean read depth of field eDNA samples, mesocosm eDNA samples, and tissue samples. | Page 6 |
| **Figure S5.** Total per-sample and per-locus read depth for eDNA samples in each mesocosm density treatment. | Page 7 |

**Table S1.** Summary information for 35 autosomal microsatellite loci used in the study, including the primer name, repeat motif, number of motif repeats, forward and reverse primer sequences, multiplex number, and allelic richness in genotyped round goby tissues. Denoted loci (†) were excluded from analysis due to poor amplification or deviation from Hardy-Weinberg equilibrium.

| Primer | Tetramer repeat | Repeat count | Forward sequence (5’-3’) | Reverse sequence (3’-5’) | Multi-plex | Allelic richness |
| --- | --- | --- | --- | --- | --- | --- |
| Nmel140 | AGAT | 11 | ATGTGCTGGATTGTCTCATTGG | AGCTTGTTAATATGGCACGGTG | 4 | 11 |
| Nmel155† | ATCC | 15 | ACATAACTTGGTGCTCATTCTGG | GGTGCAATGTGATTTGGGCAG | 4 | 3 |
| Nmel185† | AGAT | 12 | ATGTGAGACTATTTCCTTCGGG | TTTCCTCCTCTGGCTTTGTGTG | 4 | 2 |
| Nmel248† | AGAT | 13 | ACTGAATGACAACTGAATTTCCC | TCATGTTAAATTCAGATCCAGGTC | 1 | 21 |
| Nmel262 | ATCC | 10 | CGACAGCTGTAGTTCAACTCAC | AAACGGACAAGTGGAAGAAG | 1 | 7 |
| Nmel299 | AGAT | 11 | TTATGCTCACTACTGCCACTTC | AGGAAACAAGGAAGGGACAAAC | 1 | 12 |
| Nmel32 | AGAT | 11 | CTCCAAACCTTCTTACTGCTGC | AACCCTCACCTGAATCAAGCAC | 4 | 4 |
| Nmel344 | AGAT | 14 | GCTATAATGTTGTGTGCAGTGC | ACACTGCCTTAATGATTCAAAGG | 5 | 9 |
| Nmel351 | AGAT | 16 | TTTGGTCACTGGAATTACAATACG | CATATTGGCTTCAGTAGATGTTTG | 1 | 12 |
| Nmel361† | AAAG | 12 | CGAGGATCCTGGCAATTGGG | CAACTTTCGGTGCAAACATGAC | 5 | 8 |
| Nmel363 | AAGT | 10 | TCAAAGAGACCTAAGCCAGTCC | CTGTTTGGCTCGGACAACATG | 1 | 9 |
| Nmel403 | AGAT | 11 | GTCTTGCTGCATAAACCAAAGC | GGACCGATCTGTATATAGGAGCC | 5 | 10 |
| Nmel405 | AGAT | 18 | TCACAGCTAAGACCAACAACC | AATGGACTAACAGTTCGCTAC | 5 | 15 |
| Nmel411 | ATCC | 15 | GGAAACAAGGTCGCAGGTAATG | CAGCAACGGACAAGTGGAAG | 2 | 8 |
| Nmel422 | ATCC | 11 | CTCCCATCTCCCATCCAAGTAC | TACAAAGCGCGATATCAGTTGG | 2 | 3 |
| Nmel505 | AGAT | 10 | CCCACTTCAATGTTCTGCACTC | GCTTTCACCTCATTCATACAGC | 5 | 5 |
| Nmel549 | AGAT | 11 | TGCATGATATAGGACCTTTAAAGC | TGGACTGTAAGCCATAATCTTC | 6 | 11 |
| Nmel625 | ATCC | 12 | CAGACAAAGAGCGGTTCAGAAG | TGATGGTGGGTGAAGTACATGG | 6 | 10 |
| Nmel660 | ATCC | 12 | CTGGACACAAGCTGCAAGTG | CTATGTGATTTGGGCAGCTCC | 2 | 2 |
| Nmel726† | AAAG | 11 | CTCTCAGCGTATACAGAGGCC | CAGGTCCACTAATTCGATGTCC | 2 | 8 |
| Nmel729 | ACAG | 13 | ACAAGTGCATTAGTGTCATGGG | AAGGCCTAACAAACAGCAGATG | 2 | 5 |
| Nmel746 | AAAG | 17 | GTTTATGCCCTGGACATCTGC | GCTCGAGTCTTAAATGCAGATTG | 6 | 6 |
| Nmel810 | ATCC | 12 | AACACAGCGTCAAATCTCTCAG | ATTACTAGTAGGTGACGGGTCG | 7 | 7 |
| Nmel815 | AGAT | 15 | GGGTGTATTGTCTAGTCTCTGG | CAGGCTCATGTTAAGGGTTCAG | 6 | 12 |
| Nmel821 | ACAT | 11 | CCACCAATCATGTTACACAGGG | AGTTCAGAGGCAGCCAGTTG | 7 | 7 |
| Nmel89 | ACAT | 10 | TCTACTTCAGTTGGCTATGATTC | TAAGAGCAGGCTTAAGACCCAC | 7 | 6 |
| Nmel914 | AGAT | 12 | TTGTGGACAAGGGCTGAACAG | CTATACGCACAGCTTCCTCACC | 3 | 6 |
| Nmel990 | AGAT | 13 | AAATGCTGTTATACTGAGGCGG | CTCAGGGCTCCAACTACTCC | 3 | 12 |
| Nmel994 | AGAT | 11 | CTCTCTGACATGCTCCAAGGAC | TTCTCAATAACTTTATCTCGGACC | 3 | 11 |
| Nmel1103† | AAAG | 13 | GCCAAGTCCTATCTCGCAATG | CGCCCTCGGTACTGTTATAAAG | 7 | 19 |
| Nmel1132 | AGAT | 10 | TCCTGGATGAACACTACAAGGC | AGGACGTTTCGCTTTGATCTTC | 7 | 12 |
| Nmel1462 | AAAG | 10 | CGATACCCAATATAGCGGACGG | AGTCAGAAGGAAACACATGCAG | 5 | 8 |
| Nmel1486 | ATCC | 13 | TTCCCATTGTAACAGCAGAGAG | AATGTGATTTGGGCAGCTCG | 6 | 14 |
| Nmel1531† | AAAC | 10 | TGTAACAGGAGCCAAATCAGATG | GCCCTGGGTACAAACTATCTTG | 7 | 17 |
| Nmel1566 | AGAT | 14 | ATTTCGGATCCTCGTATCTGAC | AATGATATGGGAAATGCGAGCG | 3 | 19 |

**Figure S1**. (A) Sites of round goby (*Neogobius melanostomus*) sampling in Cayuga Lake, New York, USA for the mesocosm experiment and field trial. Environmental DNA samples from Cayuga Lake were collected at the field trial site. (B) We determined the round gobies collected from the two sites are panmictic, as the optimal number of clusters indicated by the lowest value of Bayesian Information Criterion (BIC) is 1.

­­**Figure S2.** Correlation of allele read depth between duplicate eDNA samples per mesocosm. Mesocosms consist of 3 replicates (A, B, or C) of each density treatment (1, 3, 5, or 10 fish). Pearson’s correlation coefficient (r) is indicated in each panel. Colors represent each of 28 loci. Diagonal lines represent a 1:1 relationship between eDNA sample 1 and sample 2 read counts.

­­**Figure S3.** Correlation of allele read frequencies between duplicate eDNA samples per mesocosm. Mesocosms consist of 3 replicates (A, B, or C) of each density treatment (1, 3, 5, or 10 fish). Pearson’s correlation coefficient (r) is indicated in each panel. Colors represent each of 28 loci. Diagonal lines represent a 1:1 relationship between eDNA sample 1 and sample 2 read frequencies.

**Figure S4.** (A) Total reads per sample and (B) total reads per locus across all alleles in 28 loci. Sample types include the three replicate field eDNA samples, 24 mesocosm eDNA samples, and tissues sampled from 73 individuals in the experiment.

**Figure S5.** (A) Total reads per sample and (B) total reads per locus across all alleles in 28 loci for eDNA samples in each mesocosm density treatment (1, 3, 5, or 10 fish).
